# Selected Consumer Behaviours in the Bread Market: Does Dietary Fibre Labelling Influence Consumer Decisions? A Preliminary Study

**DOI:** 10.3390/nu18040587

**Published:** 2026-02-11

**Authors:** Marta Sajdakowska, Dagmara Gniotek, Jerzy Gębski

**Affiliations:** 1 Department of Food Market and Consumer Research, Institute of Human Nutrition Sciences, Warsaw University of Life Sciences (WULS-SGGW), 159C Nowoursynowska Street, 02-776 Warsaw, Poland; jerzy_gebski@sggw.edu.pl; 2 P.P.H.U. “PIEKARZ S.C.”, Włodzimierz Gniotek, Dagmara Gniotek, Lipiny 29, 92-701 Łódź, Poland; kasia_gniotek@wp.pl

**Keywords:** consumer, bread, fiber, bread with increased fiber content

## Abstract

**Background/Objectives**: The current study aimed to identify consumer segments based on bread-choice motives and to examine (1) differences between segments in terms of the information provided on product labels, including information on the bread label, and (2) consumers’ perception of bread enriched with fibre, as well as the importance of fibre in the diet. **Methods**: Data were collected in 2025 using a computer-assisted web interview (CAWI) on a sample of 289 respondents. A k-means clustering segmentation method was applied, resulting in four consumer segments: “Enthusiasts”, “Ultra-Involved”, “Involved”, and “Neutral”. To quantitatively determine the impact of selected factors on the importance of information provided on product labels, a logistic regression model was developed. **Results**: The “Enthusiastic” consumers expressed the most positive opinions regarding most types of information presented on bread labels and supported the addition of fibre to bread to enhance its health benefits. Membership in the “Ultra-Involved” segment reduced the likelihood of paying attention to fibre information on the label by approximately 54% compared with the “Enthusiastic” segment. **Conclusions**: The effectiveness of information provided on food labels depends on the clarity and comprehensibility of the content, as well as on consumers’ knowledge and motivation to use such information. These findings emphasise the importance of label designs that support efficient information processing, even among highly motivated consumers. Communication strategies for fibre-enriched products should address both health benefits and sensory concerns, particularly among consumers sceptical about taste.

## 1. Introduction

Bread has been a staple of the human diet for thousands of years. Over time, a variety of bread types have emerged, adapted to local customs, ingredients and climatic conditions [1,2,3]. Although bread consumption remains high in many countries, especially in Europe, where according to data from the Atlantic Area Healthy Food Ecosystem (AHFES), the average consumption of bread and bakery products is around 57 kg per person per year, in some countries such as Germany and Austria consumption reaches as high as 80 kg, while in the United Kingdom and Ireland it does not exceed 50 kg [4]. Despite relatively high consumption levels, there has been a gradual decline in interest in bread, driven by factors such as the growing popularity of low-carbohydrate diets, negative beliefs about bread’s impact on body weight, and the perception that it is less healthy than other products [5]. A downward trend is also visible in Poland—according to Ujwary (2023), monthly bread consumption decreased from 3.31 to 2.67 kg per person between 2017 and 2021 [6].

Contemporary bread-production technologies focus on adapting bread to changing consumer needs. One of the main areas of development is enriching bread with functional ingredients such as fibre, protein, and antioxidants, thereby improving its nutritional value [7,8]. In addition, a growing number of manufacturers are striving to develop formulas with simplified, natural ingredients that exclude chemical additives, aligning with consumers’ preferences for clean-label bread [9].

Dietary fibre, one of the key health-promoting components of the diet, plays an important role in maintaining proper digestive-system function and preventing lifestyle diseases [10]. Moreover, the beneficial effects of dietary fibre on human health cover many aspects, including lowering LDL cholesterol levels and stabilising carbohydrate metabolism [8,11]. Regular fibre intake is also important in preventing chronic diseases. A review by Zhang et al. (2025) showed that a 10 g increase in daily fibre intake is associated with a 7% reduction in the risk of cardiovascular disease [10]. Fibre also has a beneficial effect on cognitive function and mental health [12]. Research also shows that regular consumption of fibre can improve sleep quality, mood and even memory in older adults [13,14]. Fibre—especially soluble fibre—can increase the production of B vitamins by commensal bacteria living in the large intestine [15].

According to current recommendations from the World Health Organisation (WHO, 2023), adults should aim for at least 25 g of dietary fibre per day to support proper digestive function and reduce the risk of chronic diseases [16]. Similar guidelines are presented by the Harvard T.H. Chan School of Public Health, which recommends a daily intake of 25–35 g of fibre, highlighting its role in controlling blood sugar, lowering cholesterol and supporting heart and gut health [17]. However, it should be noted that the European Food Safety Authority (EFSA), in its 2022 scientific report on nutrient profiling and food labelling, emphasises that fibre intake in most European populations remains below recommended levels, which may have negative health consequences and requires educational measures and product reformulation [18]. In practice, however, these values are rarely achieved—in the United States, the average daily fibre intake is only 16–17 g, which is about 50% of the recommended amount [19]. The situation is similar in Europe—in Switzerland, for example, the average fibre intake is 18 g per day [20].

To address this deficiency, dietary-fibre fortification has become increasingly common in foods such as bread, breakfast products, and snacks. Increasing fibre intake is now a key objective of public-health policy; as such, fortification provides measurable health benefits [21,22].

A variety of factors influence consumers’ choices when it comes to bread, including taste, nutritional value, price and availability. Studies show that consumers are more likely to choose wholemeal and fibre-enriched bread if it is associated with a healthy lifestyle and is readily available [23,24,25]. Price remains an important factor—consumers are less likely to buy functional products if their cost significantly exceeds the price of traditional bread [26,27]. However, perceived health benefits and the presence of nutrition claims on labels may increase the acceptance of fibre-enriched products, even if they are more expensive [28,29]. Furthermore, increased health awareness and the growing popularity of functional foods indicate that the future of the bread market will be based on innovative products tailored to consumer expectations [20,30,31,32]. Moreover, food labelling can be an effective way to encourage consumers to make healthy choices in the food market [33,34,35,36,37,38,39,40,41,42,43]. Therefore, it is necessary to monitor consumer preferences regarding the presentation of information on packaging and labels [42].

However, the effectiveness of the information included on the packaging depends largely on its clarity and presentation [39,44], especially in view of the increasingly complex content provided on labels. In addition, consumers refrain from reading the information on packaging due to its volume, lack of time and a general lack of interest in the information on food labels [43]. It should also be borne in mind that consumers may not use the nutritional information on the label, as processing it requires time, commitment and effort [45]. According to consumers, the information on labels is difficult to understand [44], and the font size and poor contrast between the text and background make it challenging for them to read [46]. Furthermore, sometimes it is not entirely clear whether existing labelling systems enable consumers to make more informed food choices [47,48,49].

Generally, EU legislation sets out the rules governing food labelling [50]. Polish national law states that bread, whether prepacked or sold loose, must be labelled in a reliable, clear and non-misleading manner. The main legal basis is the Regulation of the Minister of Agriculture and Rural Development of 23 December 2014 on the labelling of specific categories of foodstuffs. In the case of bread sold without packaging, mandatory information (including the product name, list of ingredients and producer’s details) must be made available at the point of sale; for example, on a display board or price list. Prepacked bread must additionally comply with general labelling requirements, including the declaration of allergens, net weight and the date of minimum durability [51].

With regard to fibre, EU legislation [50] does not require fibre information to be labelled. However, this information sometimes appears on food labels [52,53].

The wide range of products available on the cereal market in Poland, the preference for products with modified composition, as well as growing interest in healthy lifestyles, including the search for information on the health benefits of food, encourage research into perceptions of the role of fibre in the diet and of label information. However, despite the growing availability of fibre-enriched cereal products, including bread, there is limited empirical evidence on how consumers understand and interpret information about food products, particularly fibre-related information presented on food labels. Therefore, the current study aimed to identify consumer segments based on bread-choice motives and to examine (1) differences between segments in terms of the information provided on product labels, including information on the bread label, and (2) consumers’ perception of bread enriched with fibre as well as the importance of fibre in the diet.

## 2. Materials and Methods

### 2.1. Data Collection Process

This cross-sectional study was performed using an online survey. An online questionnaire was designed using Google Forms. The main study was preceded by a pilot study among 20 respondents aged 18–40 who purchased food and reported consuming bread. This stage enabled us to verify the correctness and understanding of the questions. Prior to the survey, all respondents were assured that the data and opinions gathered would be kept confidential and used for research purposes only. For the main study, convenience sampling was used. The survey distribution was mainly via email invitations, social media, and Internet forums in February–April (spring) 2025. At the outset of the study, recruitment was planned to last approximately 3 months to obtain a sample of 300 adult participants who consume bread at least 1–2 times per week and who declared participation or co-participation in food purchasing. Approximately 4% of the participants were recruited via a one-time email invitation sent to the staff mailing list of a higher education institution in Warsaw. The remaining 96% were recruited through four recruitment waves conducted at three-week intervals by posting the study invitation (including a link to the survey) on Facebook, six online forums dedicated to food and nutrition, and four forums focused on physical activity and body care. For this purpose, appropriate information was provided to help participants decide whether to participate in this research study. In the second, third, and fourth waves, the invitation included an additional message specifying that only individuals who had not previously participated in the study should take part. In the final sample, seven participants reported not consuming bread, and four did not meet the age eligibility criterion. In the end, 289 people were qualified for the study sample. 

### 2.2. Description of Questionnaire

The questionnaire used in the study was divided into several thematic blocks, each designed to collect data on various aspects of consumer perceptions and choices regarding bread, including fibre-enriched bread. Most of the questions were in the form of a five-point Likert scale (1—‘strongly disagree’/‘not important’, 3—‘no opinion’/‘neither important nor unimportant’; 5—‘strongly agree’/‘very important’).

With regard to the importance of factors influencing the purchase of bread (question 5), respondents were asked the following: How important are the following factors to you when buying bread? (1 = not important at all, 3 = neither important nor unimportant, 5 = very important).

The following aspects were assessed: overall appearance, product composition, energy value, taste, aroma, colour, price, crispiness, fluffiness, producer/brand, availability, freshness, bread packaging, best-before date, personal preferences and/or those of family members, bread additives (grains, bran), quality mark, place of purchase, seller’s opinion, information on the packaging, knowledge of the product.

With regard to dietary habits, selected statements available in the literature were used [54], and previously used in studies among Polish consumers were applied [24]. The following question was asked: We would like to learn more about your eating habits. To what extent do you agree with the statements below? (1—strongly disagree, 3—neither agree nor disagree, 5—strongly agree). The following statements were included: It is important to me to feel that I am obtaining good quality for my money; The information on the product packaging is very important to me; I compare the information on product labels before deciding which product to choose; I compare labels to choose products with the highest nutritional value.

Regarding limitations/barriers related to reading information on labels, the following question was asked: To what extent do you agree with the following statements regarding barriers to reading labels on bread (1—I agree to a very small extent, 3—I neither agree nor disagree; 5—I agree to a very large extent). The responses included the following statements: Reading labels takes a lot of time; Labels are printed in a very small font, which can make them difficult to read; The language used to describe the product on the label is difficult to understand.

With regard to the importance of information on bread labels, the following question was asked: How important are the following pieces of information on bread labels to you? (1—completely unimportant, 3—moderately important, 5—very important). The following factors was included: product name, producer, weight, price, product composition, fibre content, best-before date, health information, and quality mark.

Regarding the dietary fibre as a food ingredient, statements available in the relevant literature have been taken into account [55,56]. The following question was asked: To what extent do you agree with the following statements about fibre as a food ingredient (1—I completely disagree, 3—I agree to some extent, 5—I strongly agree). The answers included the following statements: Fibre satisfies hunger; Fibre accelerates the movement of food through the intestines; Wholemeal bread is a good source of fibre; Fibre helps maintain proper blood cholesterol levels; The amount of fibre consumed should be controlled.

Opinions on cereal products with added fibre items used in the research among Polish consumers [24] were included in the questionnaire. The following question was asked: To what extent do you agree with the following statements concerning cereal products with added fibre (1—I do not agree at all, 3—I agree to a moderate extent, 5—I agree to a very large extent). The proposed statements included the following: There is a need to add fibre to cereal products; Adding fibre to cereal products deteriorates their taste.

The questionnaire included questions on socio-demographic characteristics, including questions on gender, age and education.

### 2.3. Statistical Analysis

A preliminary analysis of variables was conducted, and variables were selected for segmentation purposes. Twenty-one variables determining the importance of factors when purchasing bread, ‘How important are the following factors to you when purchasing bread,’ were subjected to factor analysis with varimax rotation in order to reduce the number of dimensions. Ultimately, 19 variables were used in this analysis. Five factors, each with a logical definition, were identified (Table 1).

The correctness of the factor analysis was confirmed by Kaiser’s Measure of Sampling Adequacy, which yielded an MSA value of 0.729, an acceptable result. The total variance explained by the factors was 51.56%. The variables obtained in this way, which included factor loadings, were used for segmentation and first assigned to quintile groups (1–5) for easier interpretation. A preliminary analysis was performed using a hierarchical method to determine the number of clusters. The number of clusters was selected based on a dendrogram, and their selection was confirmed by CCC (Cubic Clustering Criteria) statistics. The obtained average cluster values were used as initial ‘seeds’ in the non-hierarchical k-means method. Ultimately, the results obtained using the non-hierarchical k-means method were utilised in the study. The four segments obtained were evaluated for the level of separation between them based on the ANOVA test, which confirmed the correctness of the selection. The clusters developed were profiled, using analysis of variance for quantitative variables and the Chi^2^ independence test for qualitative variables. All analyses were performed using the SAS 9.4 statistical package at a standard significance level of α = 0.05. To quantitatively determine the impact of selected factors on the importance of information on product labels, a logistic regression model was developed, with the label information’s importance as the dependent variable. For the purposes of dichotomisation, the variable on a 1–5 scale was grouped: (1 + 2 as “unimportant”—N = 96 and 3 + 4 + 5 as “important” N = 193). A high level of the dependent variable was predicted in the model. The model’s explanatory variables were selected statements about the fibre content of bread and cluster membership.

An analysis of the motives for choosing bread showed that in the first segment, the two most important motives were practical and nutritional ones. In the second segment, however, the most important motives were nutritional ones and those directly related to purchasing (i.e., the Direct Shopping Motives). For consumers in segment 3, practical and nutritional motives were the most important (as in segment 1). For consumers in segment 4, the two most important motives were Direct Shopping Motives and practical motives, while nutritional motives were the least important in the choice of bread (Table 2). To facilitate identification, the following names were assigned to the individual segments: Enthusiasts (1), Ultra-Involved (2), Involved (3), and Neutral (4).

## 3. Results

### 3.1. Description of the Sample and Clusters

An analysis of the sample, taking into account socio-demographic variables, reveals that women comprised the majority of the study group, and nearly half of the respondents had higher education. Among Enthusiasts, women prevailed, with more than half holding higher education, while almost 40% had completed secondary education. In the Ultra-involved segment, more than half were consumers with the highest level of education, and a quarter were people with secondary education.

In contrast, in the Involved segment, over two-fifths were consumers with higher education, and slightly over one-third had vocational education or lower. In the Neutral segment, the percentages of women and men were similar (51.4% and 48.6%, respectively), as were the percentages of people with the lowest and highest levels of education (40.3% and 37.5%, respectively) (Table 3). Approximately 65% of the consumers came from cities with a population of 50,000 inhabitants or more (Table S1).

### 3.2. Selected Eating Habits in Consumer Opinion

With regard to selected behaviours related to food and nutrition, consumers from the Enthusiasts segment emphasised to a significantly greater extent than other segments (Ultra-Involved, Involved and Neutral segments) that it is vital for them to feel that they are obtaining good quality for a specific price (It is important for me to feel that I am obtaining good quality for my money). Neutrals agreed with this opinion to a significantly lesser extent than the other three segments. In the case of opinions on the importance of information on packaging (Information on product packaging is very important to me), as well as the role of labels in food selection (I compare the information on product labels before deciding which product to choose) and the role of labels in choosing the product with the highest nutritional value (I compare labels to choose products with the highest nutritional value), people from the Enthusiasts segment emphasised that this was important to them to a significantly greater extent compared to the other three segments (Table 4).

### 3.3. Opinion on Information on the Food Labels

With regard to the legibility and manner of presenting information on food labels, consumers in the Enthusiasts segment agreed to a significantly greater extent than other segments with the statement that ‘Reading labels can be time-consuming’. The Neutral segment agreed with this opinion to the least extent (Table 5).

Regarding the information provided on bread labels, the two most important pieces of information, as proposed in the study, were considered to be the product composition and the best-before date. Their role was significantly more appreciated by respondents from the Enthusiasts segment, while the best-before date was significantly less important for the Neutral segment. The majority of the other information was also significantly important to Enthusiasts, while information on the producer was significantly less important to Neutrals compared to other segments (Table 6).

### 3.4. Fibre as a Food Ingredient and Fibre Labelling from a Consumer Perspective

With regard to the role of fibre in the diet, consumers in the Enthusiasts segment agreed to a significantly greater extent with the following opinions: Fibre satisfies hunger; Fibre accelerates the movement of food through the intestines; Wholemeal bread is a good source of fibre; and, The amount of fibre consumed should be controlled compared to other segments (Table 7).

Each one-point increase in the opinion that wholemeal bread is a good source of fibre resulted in a 9% rise in the likelihood of increasing the importance of information regarding fibre content on the label (OR: 1.090; 95% CI: 1.03–1.31).

A 1-point increase in the opinion that adding fibre to cereal products deteriorates their taste resulted in a 16.5% rise in the likelihood of increasing the importance of information regarding fibre content on the label (OR: 1.165; 95% CI: 1.01–1.35). Belonging to the Ultra-Involved cluster reduced the likelihood of increasing the importance of fibre-content information on the label by 54.1% (OR: 0.459; 95% CI: 0.18–0.98) compared to the Enthusiasts cluster (reference level). In the case of the Involved cluster, the likelihood was reduced by 68.3% (OR: 0.317; 95% CI: 0.12–0.82), and for the Neutral cluster by 67.7% compared to the reference cluster (OR: 0.323; 95% CI: 0.12–0.86) (Table 8).

## 4. Discussion

The analysis of the survey data revealed four distinct consumer segments, with Segment 1 showing the highest relative proportion of women and individuals with secondary or higher education.

### 4.1. The Importance of Selected Socio-Demographic Characteristics in Food Market Choices

The survey indicates that women attach greater importance to health than men, including proper nutrition [57,58]. Additionally, individuals with higher levels of education tend to have a better understanding of nutrition and select products that are more beneficial in terms of nutrition [59]. The profile of the Enthusiast segment may partly reflect underlying socio-demographic characteristics rather than purely motivational differences. Previous research consistently shows that women and individuals with higher education levels demonstrate greater nutrition knowledge, stronger health consciousness, and more frequent use of nutrition labels when making food choices. These groups are also more likely to engage with health-related information on food packaging and to interpret such information in line with dietary guidelines [60,61]. Gender differences in food choice have been widely documented, with women typically showing higher concern for health, dieting, and nutritional quality than men [62]. This may partly explain the stronger label engagement observed among Enthusiasts, particularly if this segment is composed of female respondents. Similarly, higher educational attainment has been associated with better comprehension of nutrition information and more frequent use of front-of-pack and back-of-pack labels [63,64]. Moreover, consumers with greater health-awareness and information-processing skills are more responsive to interpretive label formats and sustainability-related cues, which further reinforces their tendency to use labels as a decision-making tool [65,66].

### 4.2. Consumer Perceptions of the Readability and Usability of Packaging Information and Opinion Regarding Chosen Information on the Bread Label

Analysis of the obtained results showed that opinions on the importance of information on food labels received an average rating on a 5-point scale. The results of the study, in which the importance of information on food labels was rated as moderate, are consistent with other scientific findings. Although consumers often declare that they pay attention to the information on packaging and find it helpful when choosing products, the degree to which they actually use it is usually moderate rather than high [67,68,69].

An analysis of our own research revealed that the legibility of food labels was rated as average and that reading labels took a considerable amount of time for the respondents. It should also be emphasised that this opinion was most strongly shared by those belonging to the Enthusiasts segment and least strongly by those belonging to the Neutrals segment. Although it may seem counterintuitive that the “Enthusiast” segment reported nutrition labels as more time-consuming than the “Neutral” segment, this pattern is consistent with established evidence on consumer information processing. Research shows that some consumers, particularly those with stronger health goals and higher food or nutrition involvement, tend to allocate more attention and cognitive effort to nutrition information, which increases both objective processing time and subjective effort [60,70].

Some studies demonstrate that meaningful use of nutrition labels involves selective viewing of multiple components (e.g., nutrition facts, ingredients, claims), and that more engaged consumers exhibit longer fixation times and more extensive visual scanning patterns [70,71]. This deeper engagement reflects systematic information processing, rather than the superficial or heuristic use of labels, and therefore naturally entails higher perceived time-costs.

From a cognitive perspective, when health goals are salient, consumers are more likely to engage in effortful, goal-directed processing instead of relying on simple cues such as brand or price. Such systematic processing requires more time and mental resources, particularly in complex choice environments [65,66].

In addition, the growing complexity of food labels may contribute to the perceived time burden. Experimental research indicates that increased nutrition information can lead to information overload, reducing comprehension and increasing cognitive strain—even among highly motivated consumers [71,72]. Thus, Enthusiasts may experience greater “friction” because they attempt to process more label content in detail. Finally, heightened attention to labels can also reflect a risk-management strategy, where consumers carefully verify product attributes to avoid undesirable outcomes (e.g., poor taste, unhealthy composition). This aligns with evidence that nutrition knowledge and health concerns are strong predictors of label-use intensity [65]. In some consumer profiles, such vigilance is also associated with orthorexia-related tendencies, which have been linked to more frequent use of ingredient lists and nutrition facts panels [73]. A meta-analysis by Shangguan et al. (2019) indicates that food-label information can moderately influence consumer choices and support healthier eating, including lower fat intake and higher vegetable consumption. Similarly, Storz (2023) found that frequent label readers consumed more fibre and were more likely to meet dietary recommendations than infrequent readers. However, label use remains context-dependent and is mainly driven by nutritional knowledge, health motivation, and uncertainty about product composition or health effects. Overall, labels play a supportive rather than decisive role in consumer food choices [61,67,69,74]. Evidence from South Korea and Bangladesh shows that, although consumers increasingly declare reading food labels, their actual understanding and use of this information remain limited and context-dependent. The presence of information alone does not ensure its effective use, with time constraints and consumer characteristics playing a key role. Moreover, simplified, visual front-of-package labels are perceived as easier to understand and less cognitively demanding than traditional text-based formats [75,76,77]. This suggests that the average rating of label legibility obtained in our own research may result not only from the characteristics of consumers themselves, but also from the predominant way of presenting information on packaging. Taken together, the higher perceived time cost reported by Enthusiasts should not be interpreted as a weakness of the segment definition, but rather as an indication of deeper, more effortful engagement with label information. Importantly, this finding highlights a practical implication for label design: even the most motivated consumers may experience cognitive friction when labels are complex, underscoring the need for clearer information hierarchy, simplification, and user-friendly presentation formats.

An important factor differentiating the way labels are used is also the level of nutritional knowledge and awareness. The literature suggests that individuals with a higher level of knowledge are more likely to read labels frequently and pay closer attention to them, while simultaneously reporting greater difficulties in interpreting them [78]. These results are consistent with observations from our own research, in which segment 1 could represent consumers who are more aware and more involved in analysing information.

Regarding the information on bread labels, it was noted that the most important information for the majority of respondents was the product composition and best-before date, which seems particularly understandable in the case of bread. Consumers in the first segment considered both pieces of information to be significantly more important than those in the other segments.

The literature studies indicate that consumers value simple product composition (a short list of ingredients), while the more complex the composition, the less likely they are to choose the product [79]. The best-before date strongly influences the assessment of the risk associated with the consumption of a given food product, which limits the acceptance of bread whose best-before date is approaching, even if its sensory quality is preserved [80].

In the study by Heenan et al. (2009), freshness is one of the strongest determinants of bread selection. Consumers use indirect indicators of freshness (softness, smell, appearance) rather than just date information. The best-before date serves as a risk signal rather than a guarantee of quality. The freshness of bread is assessed mainly heuristically, and the best-before date plays a supporting role, especially in the case of packaged bread [81].

### 4.3. Fibre as a Food Ingredient from a Consumer Perspective

In our own research on fibre as a food ingredient, the opinion that fibre satisfies hunger and accelerates the movement of food through the intestines was most widely agreed upon. It should be noted that consumers in the Enthusiasts segment agreed with this statement the most. ‘Improving bowel movements’ was also identified as one of the basic benefits of fibre in studies by other authors, while ‘reducing energy intake’, which may refer to satisfying hunger, was less frequently identified by the respondents [82]. However, the literature review results indicate a need to increase consumer knowledge of dietary fibre through education (e.g., highlighting nutritionally beneficial types of food and examples of foods) [83]. It should be emphasised, however, that in the light of the literature, people from different groups, in terms of age, gender, education, living environment, or country, demonstrate different perceptions and information about dietary fibre [84].

Both those who considered wholemeal bread to be a good source of fibre and those who were concerned about the addition of fibre to cereal products due to a deterioration in taste were more likely to consider information about fibre to be important, which may contribute to their seeking this information on the label. The results of previous studies have shown a positive association between information on fibre addition on the label and the acceptance or willingness to purchase bread with fibre [85,86,87]. The literature suggests that, in the opinion of consumers, the addition of fibre may compromise the taste of the final product, and consumers are not always willing to accept it [85]. Moreover, from the consumer point of view, unhealthy foods are tastier than healthy foods [88].

Compared to other segments, individuals in the Enthusiasts segment attached the greatest importance to information about fibre content on the labels of bread and cereal products. The literature studies indicate that certain consumer groups have demonstrated a greater interest in product information, including nutritional details. In the study by Grunert et al. (2010), those interested in healthy eating and nutrition claims were more likely to search for information on food labels [60]. Research indicates that some consumers actively use packaging claims in their market choices, while others disregard or distrust them [60,89].

The regression result, indicating that consumers who believe that fibre worsens taste are more likely to check nutrition labels (OR = 1.165), suggests a risk-reduction mechanism rather than a purely health-seeking motivation. Previous research shows that consumers often use labels not only to pursue health benefits, but also to avoid negative product experiences, such as poor taste, undesirable texture, or unfamiliar ingredients [60,65]. When taste concerns are salient, consumers may engage more actively in label checking to manage uncertainty and reduce the perceived risk of dissatisfaction.

This interpretation is consistent with studies showing that taste expectations strongly influence food choice and that consumers frequently consult ingredient lists and nutrition information when they anticipate potential sensory drawbacks [90,91]. Fibre enrichment has been shown to compromise the sensory acceptance of bread and other cereal products, particularly with respect to texture and overall palatability [92,93]. This may increase perceived product uncertainty among sceptical consumers, who, in turn, are more likely to engage in compensatory information-seeking behaviour, such as consulting label information to reduce perceived purchase risk [94].

Moreover, label use has been shown to function as a risk-management strategy, especially for products perceived as unfamiliar or potentially unpleasant [65,95]. Consumers who expect negative sensory outcomes may therefore rely more on label information to evaluate product composition and to avoid disappointing purchases. This finding extends the literature by showing that label consultation can be motivated not only by health objectives but also by taste-related risk avoidance.

### 4.4. Strengths and Limitations

This study has several limitations. Most notably, the sample size was relatively small and based on a convenience sampling approach, which restricts the generalisability of the findings to the broader population. Future research should therefore involve larger and more representative samples and may also explore how consumers assess label information related to nutrients other than those examined in the present study. Furthermore, although information on respondents’ place of residence (urban versus rural) was collected, this variable was not included in the analytical models. As previous research suggests that bread consumption practices, trust in food products, and attachment to bread-related traditions may differ between urban and rural populations, future studies should explicitly examine these differences. The study did not collect detailed information on the point of purchase (e.g., small bakery, farm shop, supermarket, hypermarket), even though points of sale may act as trust cues independent of label information and may shape consumers’ perceptions of quality and authenticity. Moreover, the questionnaire relied exclusively on closed-ended questions, which limited the exploration of cultural, symbolic, and contextual meanings associated with bread consumption. Future research would benefit from mixed-method approaches combining quantitative surveys with qualitative techniques, as well as from a more detailed examination of the diversity and recognition of labels present in the bread market, including potential complementarities or contradictions between certification labels and private- or company-specific labels.

Moreover, although the Enthusiast segment exhibits a health-oriented behavioural profile, this pattern should be interpreted with caution. Future studies should statistically control for other key socio-demographic variables, particularly gender and education, in regression models to disentangle demographic influences from attitudinal segmentation and better assess whether Enthusiasts constitute a truly distinct behavioural group.

Importantly, this result highlights that consumer engagement with labels is multidimensional: it reflects both approach motives (seeking health benefits) and avoidance motives (preventing negative experiences). Recognising this dual function of label use has implications for label design, suggesting that clear and transparent information about ingredients and sensory-relevant attributes may help consumers manage taste-related concerns more effectively.

Despite these study limitations, we hope that our findings can provide practical implications; for example, regarding food label information, as well as more generally for the communication of information to consumers at the point of sale, including in small shops and in supermarkets and hypermarkets. Furthermore, in addition to educational campaigns on healthy eating habits, it is worth considering targeted campaigns for other age groups (i.e., young, middle-aged, and older consumers). Moreover, research focusing on the role of food labels, particularly their readability in consumer decision-making, remains highly relevant, as it provides important guidance for food manufacturers, processors, and policymakers responsible for designing and implementing food-labelling regulations.

## 5. Conclusions

Information provided on food labels, including those on bread products, plays an important role in consumer decision-making. Its effectiveness, however, depends on the clarity and comprehensibility of the content, as well as on consumers’ knowledge and motivation to use such information.

The Enthusiast segment shows a more positive attitude toward food labels than the other consumer groups. At the same time, Enthusiasts are more aware that reading and interpreting labels is time-consuming, indicating a higher level of cognitive engagement rather than passive label-use. This segment also demonstrates a better understanding of the role of dietary fibre in the diet compared with the remaining segments. In addition, consumers who believe that fibre negatively affects the taste of food are more likely to consult label information, suggesting that label use is driven not only by health-oriented motives but also by taste-related risk reduction.

These findings emphasise the importance of label designs that support efficient information processing, even among highly motivated consumers. A clear and well-structured presentation of fibre-related information may reduce the perceived time-burden associated with label reading. Communication strategies for fibre-enriched products should address both health benefits and sensory concerns, particularly among consumers sceptical about taste. From a policy perspective, these results support the need for regulatory frameworks and public-health initiatives that promote clear, standardised, and consumer-friendly presentation of key nutritional information, thereby facilitating informed food choices across different consumer segments.

Summing up, educational initiatives should be strengthened to encourage not only consumers classified as Enthusiasts but also other consumer groups to make greater use of label information, including information on the dietary role of fibre. Such initiatives should be jointly undertaken by representatives of the scientific community and the food industry.

## Figures and Tables

**Table 1:** Principal components analysis (PCA) of consumers’ use of bread selection motives; varimax rotated factor loadings percentage of explained variance (N = 289, Poland).

Bread Selection Motives	The Practical Motives Factor 1	The Marketing Motives Factor 2	The Nutritional Motives Factor 3	The Product Recognition and Quality Motives Factor 4	The Direct Shopping Motives Factor 5	Alpha
Colour	0.64439					0.840
Own preferences and/or those of family members	0.57932					
Freshness	0.5729					
Taste	0.56592					
General appearance	0.47503					
Smell	0.46736					
Producer/brand		0.65481				0.737
Seller’s opinion		0.63772				
Bread packaging		0.575				
Bread additives (e.g., grains, bran)		0.52048				
Calorific value			0.76561			0.712
Product composition			0.71583			
Best-before date			0.51279			
Product familiarity				0.57721		0.683
Crispiness				0.5469		
Fluffiness				0.52447		
Quality mark				0.49905		
Place of purchase					0.59912	0.621
Price					0.52598	

**Table 2:** Characteristics of the identified segments according to the PCA factors (motives for bread selection); the mean ratings of the segments on the classification variables.

Bread Selection Motives	1 Enthusiasts (N = 42)	2 Ultra-Involved (N = 100)	3 Involved (N = 75)	4 Neutral (N = 72)	*p*-Value
The Practical Motives	4.56 ^a^ (0.38)	3.19 ^b^ (0.87)	3.12 ^b^ (0.67)	3.32 ^b^ (0.60)	<0.0001
The Marketing Motives	3.73 ^a^ (0.75)	2.63 ^c^ (0.79)	2.90 ^b^ (0.79)	3.14 ^b^ (0.83)	<0.0001
The Nutritional Motives	4.42 ^a^ (0.49)	3.68 ^b^ (0.61)	3.12 (0.84) ^c^	2.03 ^d^ (0.50)	<0.0001
The Product Recognition and Quality Motives	4.29 ^a^ (0.52)	2.95 ^c^ (0.72)	2.88 ^c^ (0.88)	3.22 ^b^ (0.67)	<0.0001
The Direct Shopping Motives	4.25 ^a^ (0.65)	3.55 ^b^ (0.62)	1.81 ^c^ (0.53)	3.46 ^b^ (0.75)	<0.0001

Different superscripts indicate significantly different means following the ANOVA post hoc Waller-Duncan test. Standard deviations are shown in parentheses.

**Table 3:** Socio-demographic characteristics of the consumers surveyed (N = 289, Poland).

Variables	Total (N = 289)	1 Enthusiasts (N = 42)	2 Ultra-Involved (N = 100)	3 Involved (N = 75)	4 Neutral (N = 72)	*p*-Value
Gender						
Female	59.5	78.6	60.0	56.0	51.4	0.034
Male	40.5	21.4	40.0	44.0	48.6	
Age						
18–25	21.5	26.1	20.0	16.0	26.4	0.513
26–40	39.8	40.5	42.0	42.7	33.3	
41–50	22.5	16.7	27.0	20.0	22.2	
50+	16.2	16.7	11.0	21.3	18.1	
Education						
primary/vocational	27.7	7.1	21.0	36.0	40.3	0.002
secondary	24.6	38.1	25.0	18.7	22.2	
higher	47.7	54.8	54.0	45.3	37.5	

Chi^2^ test of independence, *p*-value < 0.05—differences between groups are significant.

**Table 4:** Level of agreement with statements relating to selected eating habits.

Statements	Mean	1 Enthusiasts (N = 42)	2 Ultra-Involved (N = 100)	3 Involved (N = 75)	4 Neutral (N = 72)	*p*-Value
It is important for me to feel that I am obtaining good quality for my money	3.30 (1.39)	4.12 ^a^ (0.94)	3.45 ^b^ (1.40)	3.20 ^b^ (1.35)	2.69 ^c^ (1.36)	<0.0001
The information on the product packaging is very important to me	3.12 (1.37)	3.79 ^a^ (1.20)	3.09 ^b^ (1.32)	3.04 ^b^ (1.37)	2.86 ^b^ (1.43)	0.0046
I compare the information on product labels before deciding which product to choose	3.11 (1.38)	3.71 ^a^ (1.20)	3.13 ^b^ (1.40)	3.04 ^b^ (1.44)	2.82 ^b^ (1.31)	0.009
I compare labels to choose products with the highest nutritional value	3.03 (1.40)	3.64 ^a^ (1.10)	3.11 ^b^ (1.38)	2.91 ^b^ (1.44)	2.68 ^b^ (1.42)	0.0034

Different superscripts indicate significantly different means following the ANOVA post hoc Waller– Duncan test. Standard deviations are shown in parentheses.

**Table 5:** Opinion on information on the food labels.

Statements	Mean	1 Enthusiasts	2 Ultra-Involved	3 Involved	4 Neutral	*p*-Value
Reading labels can be time-consuming.	3.22 (1.40)	3.81 ^a^ (1.19)	3.32 ^b^ (1.38)	3.25 ^b^ (1.33)	2.69 ^c^ (1.45)	0.0003
The labels are printed in very small font, which can make them difficult to read.	3.01 (1.25)	3.33 (1.24)	2.98 (1.29)	2.93 (1.40)	2.90 (1.35)	0.384
The language used in the description on the label is difficult to understand.	2.83 (1.41)	2.81 (1.45)	2.61 (1.31)	2.96 (1.47)	3.00 (1.44)	0.2479

Different superscripts indicate significantly different means following the ANOVA post hoc Waller–Duncan test. Standard deviations are shown in parentheses.

**Table 6:** Importance of information on bread labels.

Information	Mean	1 Enthusiasts	2 Ultra-Involved	3 Involved	4 Neutral	*p*-Value
product composition	3.48 (1.35)	4.40 ^a^ (0.80)	3.43 ^b^ (1.35)	3.32 ^b^ (1.36)	3.17 ^b^ (1.37)	<0.0001
best-before date	3.35 (1.46)	4.33 ^a^ (0.85)	3.4 ^b^ (1.45)	3.29 ^b^ (1.48)	2.75 ^c^ (1.44) ^c^	<0.0001
product name	3.17 (1.42)	3.79 ^a^ (1.07)	3.05 ^b^ (1.39)	3.13 ^b^ (1.51)	3.01 ^b^ (1.46)	0.0215
product mass/weight	3.22 (1.43)	3.71 (0.99)	3.16 (1.48)	3.12 (1.50)	3.11 (1.48)	0.1134
price	3.23 (1.38)	3.88 ^a^ (1.02)	3.29 ^b^ (1.44)	3.16 ^b^ (1.34)	2.85 ^b^ (1.39)	0.0013
information on fibre content	3.15 (1.40)	3.79 ^a^ (1.22)	3.11 ^b^ (1.38)	2.96 ^b^ (1.47)	3.03 ^b^ (1.36)	0.0125
producer	3.07 (1.34)	3.55 ^a^ (1.02)	3.01 ^c^ (1.40)	3.03 ^bc^ (1.33)	2.92 ^c^ (1.42)	0.0484
health information	3.09 (1.42)	3.57 ^a^ (1.17)	2.88 ^b^ (1.41)	3.16 ^ab^ (1.43)	3.04 ^ab^ (1.51)	0.0433
quality mark	2.97 (1.38)	3.67 ^a^ (1.07)	2.82 ^b^ (1.31)	2.97 ^b^ (1.38)	2.75 ^b^ (1.54)	0.0031

Different superscripts indicate significantly different means following the ANOVA post hoc Waller–Duncan test. Standard deviations are shown in parentheses.

**Table 7:** Level of agreement with statements concerning fibre as a food ingredient.

Statements Concerning Fibre	Mean	1 Enthusiasts	2 Ultra-Involved	3 Involved	4 Neutral	*p*-Value
Fibre satisfies hunger	3.35 (1.34)	4.00 ^a^ (1.13)	3.28 ^b^ (1.35)	3.41 ^b^ (1.33)	3.00 ^b^ (1.35)	0.0015
Fibre accelerates the movement of food through the intestines	3.35 (1.44)	4.43 ^a^ (0.77)	3.37 ^b^ (1.45)	2.95 ^b^ (1.37)	3.11 ^b^ (1.49)	<0.0001
Wholemeal bread is a good source of fibre	3.20 (1.43)	4.12 ^a^ (1.17)	3.07 ^b^ (1.39)	3.2 ^b^ (1.46)	2.86 ^b^ (1.40)	<0.0001
Fibre helps maintain healthy blood cholesterol levels	3.18 (1.33)	3.57 (1.13)	3.16 (1.31)	3.08 (1.25)	3.10 (1.51)	0.2044
The amount of fibre consumed should be controlled	3.14 (1.45)	4.12 ^a^ (1.04)	3.21 ^b^ (1.47)	2.81 ^b^ (1.47)	2.81 ^b^ (1.35)	<0.0001

Different superscripts indicate significantly different means following the ANOVA post hoc Waller–Duncan test. Standard deviations are shown in parentheses.

**Table 8:** Prediction of the importance of fibre-content information on the labels of cereal products and bread.

Parameter	Estimate	Point Estimate (OR)	95% Wald Confidence Limits		*p*-Value
Intercept	1.225				0.0491
Wholemeal bread is a good source of fibre	0.087	1.090	1.03	1.31	0.0457
There is a need to add fibre to cereal products	−0.107	0.899	0.78	1.04	0.1658
Adding fibre to cereal products deteriorates their taste	0.153	1.165	1.01	1.35	0.0448
Cluster 2—Ultra-Involved	−0.779	0.459	0.18	0.98	0.0451
Cluster 3—Involved	−1.149	0.317	0.12	0.82	0.0183
Cluster 4—Neutral	−1.129	0.323	0.12	0.86	0.0231
Cluster 1—Enthusiasts (ref.)	0	1			

OR—point estimate (e^β^), 95% confidence intervals.

## Data Availability

The original contributions presented in the study are included in the article, further inquiries can be directed to the corresponding author.

## Supplementary Materials

The following supporting information can be downloaded at: https://www.mdpi.com/article/10.3390/nu18040587/s1, Table S1: Sample characteristics by place of residence (N = 289).
