# Supplementary material for: Selected Consumer Behaviours in the Bread Market: Does Dietary Fibre Labelling Influence Consumer Decisions? A Preliminary Study
Source: Nutrients. 2026 Feb 11;18(4):587. doi: 10.3390/nu18040587 (PMC12943508; doi:10.3390/nu18040587)
Supplement: Supplementary file 1 [file nutrients-18-00587-s001.zip › nutrients-4101217-supplementary.pdf]

Table S1. Sample characteristics by place of residence (N=289).

Place of residence	Total	Enthusiasts	Ultra-Invlved	Involved	Neutral	p-value
Rural	20.42	40.48	23.0	12.0	13.89	0.0002
< 50,000 inhabitants	14.88	2.38	14.0	18.67	19.44	
50,000–500,000 inhabitants	45.33	26.19	46.0	44.0	56.94	
500,000 inhabitants and more	19.38	30.95	17.0	25.33	9.72	
